# Relationship between Selected SNPs (g.16024A/G, g.16039T/C and g.16060A/C) of the FASN Gene and the Fat Content and Fatty Acid Profile in the Milk of Three Breeds of Cows

**DOI:** 10.3390/ani14131934

**Published:** 2024-06-29

**Authors:** Paulina Przybylska, Marian Kuczaj

**Affiliations:** Institute of Animal Breeding, Wroclaw University of Environmental and Life Sciences, ul. Chelmonskiego 38C, 50-375 Wroclaw, Poland

**Keywords:** FASN, SNP, milk, fat milk, fatty acids, cattle

## Abstract

**Simple Summary:**

Fat is a component of milk and is an object of interest for consumers and milk producers. It consists of saturated fatty acids, which are the main group of acids present, and unsaturated fatty acids, occurring in smaller amounts in cow’s milk. The nutritional value of milk is determined by, among other things, the fat content and fatty acid composition of milk. The aim of this study was to determine the relationships of three single nucleotide polymorphisms (SNPs) and their genotypes with the fat contents and fatty acid profiles of milk from cows of breeds: Polish Red-White (ZR), Polish Red (RP), and Polish Holstein-Friesian Red-White (RW). The frequencies of the genotypes of SNPs for each cattle breed studied were also determined. The research results allowed us to identify which breed of cattle (with its specific genotype of a given SNP) produces the best milk based on the fat content and fatty acid composition of the milk. It turned out that, in the case of two SNPs, it was the ZR breed, and in the case of the third SNP, it was the RP breed. This fact can be used in the breeding of cattle to improve the quality of their milk.

**Abstract:**

Fat is an important energy and nutritional component of milk and consists of fatty acids. FASN (fatty acid synthase) is an enzyme that regulates the synthesis of fatty acids in the milk and meat of cattle. It was hypothesized that knowing the relationships between the genotypes of the tested single nucleotide polymorphisms (SNPs) and the content of fat and specific fatty acids would make it possible to improve milk quality in the selection process during cattle breeding. This study aimed to analyze the relationships of SNPs (g.16024A/G, g.16039T/C) of the FASN gene and their genotypes with the fat and fatty acid content of the milk of the following breeds: Polish Red-White (ZR), Polish Red (RP), and Polish Holstein-Friesian Red-White (RW). The SNP g.16060A/C was included in the study, although its effect on the fat composition of cow’s milk has not yet been widely studied. Milk was obtained during test milkings. SNP genotyping was performed using the real-time PCR (HRM) method. The milk from ZR and RP cows was more often characterized by a more favorable fatty acid profile than the milk from RW cows. This information can be used by cattle breeders and consumers of so-called functional food.

## 1. Introduction

Cow’s milk is a food consumed daily around the world [1]. It is considered a complete food that provides important macro- (carbohydrates, proteins, and fat fractions) and micronutrients (vitamins, minerals) in the human diet [2,3]. Among the nutrients and energy-containing components of milk, fat is the most important [4,5]. The milk fat content is 3–4% [6,7]. Its main component is triacylglycerols (TGs—98% of milk fat). The remaining part of milk fat consists of di- and monoacylglycerols, phospholipids, and free fatty acids. Milk fat consists of more than 400 fatty acids, but only 15 acids account for almost 1% or just over 1%. The remaining fatty acids are present in trace amounts [6]. The main group of fatty acids in cow’s milk are saturated fatty acids (SFAs),accounting for about 70% of the fatty acid content. On the other hand, monounsaturated fatty acids (MUFAs—about 26% of the fatty acid content) and polyunsaturated fatty acids (PUFAs—about 4% of the fatty acid content) are present in cow’s milk in much lower concentrations [7]. The de novo synthesis of the C4:0–C14:0 fatty acids and some of the C16:0 fatty acids takes place in the mammary gland. The remaining portion of theC16:0 acids and acids longer than C18 (long-chain fatty acids, LCFAs) are absorbed from the blood [8]. Some fatty acids can also be synthesized in the intestines or in the adipose tissue [7].

Fatty acids have various effects on human health; for example, SFAs such as C12:0 (lauric acid), C14:0 (myristic acid), and C16:0 (palmitic acid) have a hypercholesterolemic effect, which is caused by an increase in the level of low-density lipoprotein (LDL) [9]. Therefore, it is believed that SFAs in milk and dairy products are a risk factor for cardiovascular diseases [10,11]. One of the main acids from the group of MUFAs, oleic acid (C18:1n9), participates in lowering LDL levels and, at the same time, maintains HDL levels at a level beneficial to health [11]. Dairy products, characterized by high nutritional value, have many other benefits for human health. They have been found to have a positive effect on the control of obesity, type 2 diabetes, and Alzheimer’s disease. The consumption of dairy products also contributes to a reduction in the risk of stroke, hypertension, and colorectal, bladder, and stomach cancer [2].

Fat biosynthesis in cow’s milk is a process regulated by several genes [5,12]. The gene that regulates the *de novo* biosynthesis of long-chain fatty acids is the FASN (*Fatty Acid Synthase*) gene. It is a homodimeric enzyme that plays an important role in the overall metabolism of animals [13], especially in the process of fatty acid synthesis in adult mammals, but also plays an important role during their embryonic development [4,14]. The bovine FASN gene (GenBank accession AF285607) is 19,770 bp long and consists of 42 exons and 41 introns [15],and was mapped on the longer arm of chromosome 19 (BTA19)–19q22 [16]. FASN, being necessary for the elongation of fatty acids in the process of their *de novo* synthesis, also determines the synthesis of saturated fatty acids (SFAs) and unsaturated fatty acids (UFAs) [17]. FASN is a cytosolic enzyme that catalyzes the *de novo* formation of palmitate from acetyl-CoA and malonyl-CoA in the presence of NADPH [13,18,19,20].

Scientists have repeatedly indicated that the SNPs (single nucleotide polymorphisms) of the FASN gene, g.16024A/G (rs208645216) and g.16039T/C (rs209734560), significantly affect the fat content and fatty acid composition of the milk and meat of cattle of different breeds [15,19,21,22,23,24,25,26,27,28,29].

The study hypothesized that, knowing the significant relationships between the identified SNPs genotype variants of the FASN gene and the content of fat and specific fatty acids, it is possible to improve the quality of milk by selecting specific genotypes of individual cattle breeds in the breeding of these animals (marker-assisted selection, MAS). The identified marker allele associated with a favorable QTL allows the selection process to increase the frequency of this allele’s appearance and, consequently, contributes to the improvement of milk quality (including the improvement of its composition) [13].

The aim of this study was to determine the effect of tested SNPs (along with their genotypic variants) on the fat content and fatty acid composition of milk from three breeds of cow: Polish Red-White (ZR), Polish Red-Red (RP), and Polish Holstein-Friesian Red-White (RW). The SNP g.16060A/C (rs211379310) was also studied, although its effect on the composition of milk fat and on the fatty acid profile of cows has not yet been sufficiently studied by scientists. In addition, the genotype frequencies of each of the three tested SNPs of the FASN gene in all three studied cattle breeds were determined. The ZR and RP breeds are breeds that are covered by conservative breeding in Poland.

The research results allowed us to identify which breed of cattle (with its specific genotype of a given SNP) produces the best milk due to the fat content and fatty acid composition of the milk. It turned out that, in the case of two SNPs, it was the ZR breed, and in the case of the third SNP, it was the RP breed. The obtained results should constitute the basis for expanding research on the investigated polymorphisms of the FASN gene (also in the scope of the unknown SNP g.16060A/C) and the possibility of using them as a genetic marker supporting the improvement of the quality of cow’s milk.

## 2. Materials and Methods

### 2.1. Research Material

The research material consisted of 485 milk samples obtained from cows belonging to three breeds: Polish Red-White (ZR, *n* = 95), Polish Red (RP, *n* = 148), and Polish Holstein-Friesian Red-White variety (RW, *n* = 242). The milk was produced during the summer season (July) and was obtained during a trial milking (A4 and AT4 methods). The cows were fed with the traditional barn–pasture system (PMR—partially mixed ration in a barn with pasture season grazing). The farms (*n* = 17) from which the studied cows came were located in northeastern, southwestern, and southern Poland and were included in the assessment of the use value of dairy cattle.

Because milk samples were obtained during trial milkings (performed regularly according to a specific schedule by a qualified zootechnician), there was no need to gain approval for the procedure for obtaining research material from the Local Ethical Committee. The animals included in the study were not subjected to new stress factors.

After being collected in plastic containers, the milk samples were cooled at 4 °C and transported to the laboratory. The samples were cooled until their fat content was determined. After delivery to the laboratory, milk samples intended for DNA isolation were frozen at −20 °C until DNA isolation was carried out.

The somatic cell count (SCC) of the tested milk did not exceed 400,000/mL. This indicates that the cows in this study were in good health. The cows from which the milk was obtained were kept in good welfare conditions. The herds of cows included in the study were under constant medical and veterinary care. Moreover, the information obtained from the breeders showed that the cows were not sick and were not being administered any medications when the samples were obtained. The above information indicates that the cows in this study were in good health.

### 2.2. Milk Analyses

The fat content of the milk samples were determined using an Infrared Milk Analyzer 150 (Bentley Instruments Inc., Chaska, MN, USA) and their somatic cell count (SCC) was determined using a Somacount 150 (Bentley Instruments Inc.).

The fatty acid profile was determined using a gas chromatograph Agilent Technologies 7890A GC System with a flame-ionization detector (Santa Clara, CA, USA). Fat was extracted from milk using the Folch method [30]. Esterification was carried out with potassium hydroxide (KOH) in a mixture of methanol and hexane. Individual fatty acid esters were determined using a standard mixture of fatty acid methyl esters—Supelco^TM^ 37 (Sigma Aldrich, St Louis, MO, USA)—together with the CLA isomers cis-9, trans-11, trans-10, and cis-12 (Larodan, Malmö, Sweden). To separate the obtained fatty acid esters, an HP-88 capillary column with the dimensions 100 m × 0.25 mm × 250 μm was used. The temperature of the furnace increased at a rate of 4 °C/min starting from the initial isotherm from 100 °C to 140 °C and then increased at a rate of 2 °C/min to a temperature of 240 °C. The FAME mixture (1 μL) was injected automatically in split mode 20:1 at an injector temperature of 250 °C and a detector temperature of 270 °C. Helium was used as the carrier. Agilent ChemStation no. B.03.02. (Agilent Technologies) software was used to identify particular esters of fatty acids.

In the milk fat of the studied breeds of cows, the percentage of 28 fatty acids was determined. These were fatty acids classified as saturated fatty acids (SFAs); short-chain fatty acids with ≤6 carbon atoms in the chain (SCFAs); unsaturated fatty acids (UFAs); monounsaturated fatty acids (MUFAs); polyunsaturated fatty acids with more than 1 double bond in the hydrocarbon chain (PUFAs); medium-chain fatty acids with 8–17 carbon atoms in the chain (MCFAs); and long-chain fatty acids with ≥18 carbon atoms in the chain (LCFAs). The percentage of all identified trans fatty acids C18:1 was determined. C15:1 and C18:2n6t acids were not included in further analyses due to their very low content in the tested milk fat. The total amount of all determined SFAs and all tested UFAs was considered in this study. The sum of the total SFA and UFA content was also analyzed.

### 2.3. Isolation of Genomic DNA. SNPs Genotyping

To isolate genomic DNA from milk, the Sherlock AX kit (A&A Biotechnology, Gdańsk, Poland) was used according to the manufacturer’s instructions. The concentration and purity of DNA isolates were then determined using a Nano Drop 2000c spectrophotometer (Thermo Fisher Scientific, Wilmington, DE, USA).

Genotyping the single nucleotide polymorphisms (SNPs) such as g.16024A/G (rs208645216)—SNP 1*; g.16039C/T (rs209734560)—SNP 2*; and g.16060A/C (rs211379310)—SNP 3* in the FASN gene was planned. The mentioned SNPs are located in the 34thexon of the FASN gene [24,27,28]. (* Contractual designations of the tested SNPs were adopted).

The isolated DNA fragments were amplified using real-time polymerase chain reaction (PCR). Real-time PCR with high-resolution melting (HRM) analysis was carried out using the HRM master mix–SensiFAST^TM^ HRM kit (Bioline Ltd., Meridian Bioscience, London, UK) with the Real-time 7500 Fast thermocycler (Applied Biosystems).

The composition of the PCR mixture (20 μL) for all the tested SNPs was optimized as follows: 10 μL of Master Mix (SensiFAST HRM Mix), 1 μL of 10 μM of forward primer, 1 μL of 10 μM of reverse primer, 2 μL of DNA solution, and 6 μL of molecular water (nuclease-free). Information about the sequences of primer pairs, annealing temperature, and location and amino acid replacement for each SNP tested is presented in Table 1.

**Figure 1:**
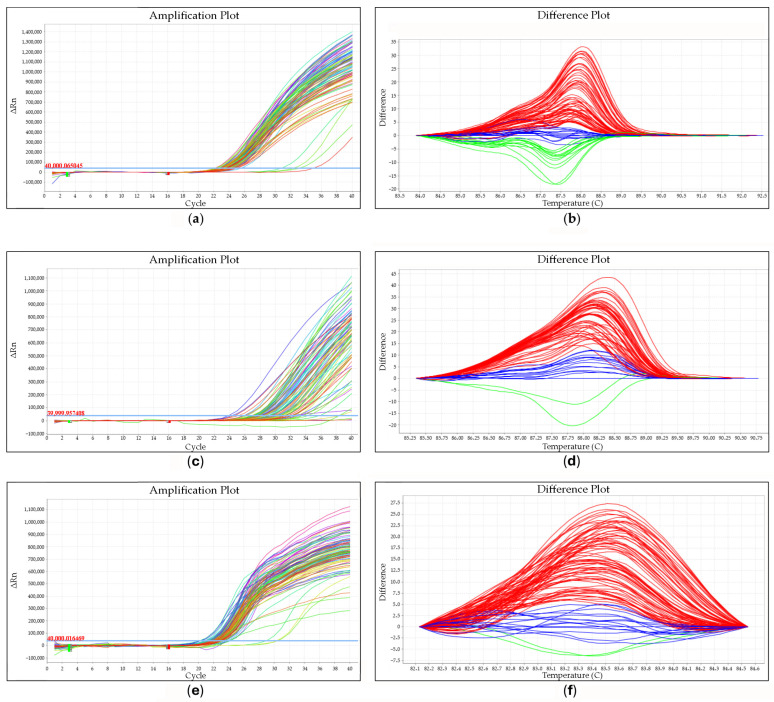
An example of the amplification diagram of the test samples for SNP 1 (**a**), SNP 2 (**c**) and SNP 3 (**e**). An example of the high-resolution melting profile (HRM) of the amplicons of the tested samples for SNP 1 (**b**), SNP 2 (**d**), and SNP 3 (**f**). (**b**): red—GG homozygotes; blue—A/G heterozygotes; green—AA homozygotes. (**d**): red—CC homozygotes; blue—T/C heterozygotes; green—TT homozygotes. (**f**): red—CC homozygotes; blue—A/C heterozygotes; AA—homozygotes.

Eva Green^®^ fluorophore was used for HRM analysis. The temperature and time conditions for the amplification (Figure 1a,c,e) were optimized as follows: initial denaturation (95 °C for 3 min) and 45 cycles of PCR (denaturation: 95 °C for 10 s, primer hybridization: 65 °C for 10 s, elongation: 72 °C for 15 s). After the elongation stage, it was possible to read the fluorescence recorded in the FAM channel. The temperature and time profile of the amplicon melting profile analysis was as follows: 95 °C for 15 s, 65 °C for 15 s, and 95 °C for 0.5/5 s. While heating the sample, fluorescence was measured. A sharp decrease in fluorescence was recorded upon the denaturation of double-stranded DNA. The recorded melting temperature was determined by the DNA length and base pair content. On the basis of the obtained profiles of high-resolution melting profiles analysis (HRM)of the amplicons, the occurrence of three genotypes in each of the studied SNPs was found (Figure 1b,d,f). The shape of the melting curve indicates a specific genotypic variant.

Amplification plots of tested samples and high-resolution melting (HRM) profiles of amplicons (Figure 1a–f) were created using 7500 software version 2.0.5 for the Real Time 7500 Fast thermocycler (Applied Biosystems).

To determine genotype variants for each tested SNP, a pattern of differential melting curves for individual genotypes was used using the protocol included with the HRM master mix: SensiFAST^TM^ HRM kit (Bioline Ltd., Meridian Bioscience).

The genotyping of SNPs involves comparing melting curves between genotype variants with different melting temperatures (T_m_), which depend on sequence variability. The different T_m_ values between the two homozygous variants are distinguished by the temperature shift in the melting curves. Heterozygotes are distinguished based on the different shapes of the melting curve [31] (Figure 1b,d,f).

### 2.4. Statistical Analysis

The analysis normality of the distributions of the variables was verified using the Kolmogorov–Smirnov test. The multivariate analysis of variance and Bonferroni’s post-hoc multiple comparison tests were used to verify the significance of the differences.

To compare the observed frequencies with the expected frequencies, assuming the null hypothesis that there was no relationship between the two variables, Pearson’s chi-square (χ^2^) test was used to confirm the Hardy–Weinberg law for the tested gene in the tested animal population. In addition to checking whether there was a relationship between the variables (chi-square statistic (χ^2^)), they also checked how strong the relationship was. Cramer’s V coefficient (Vc) was used to measure the strength of this relation. For all the analyses, a significance level of 0.05 (*p* < 0.05) was assumed. In subsequent analyses, the interactions between breed and the genotype SNP 1 were verified to determine whether they significantly differentiate the results of the analyzed dependent variables (fat content and individual milk fatty acids tested). For this purpose, analysis of variance for factorial systems was used. The significance of the differences (*p* < 0.05) and the strength of the effect (eta-squared (η^²^) fractional, Fisher’s (F) test) for the analyzed interactions were determined. After finding significant differences (*p* < 0.05) for specific dependent variables, at least between the two study groups, Bonferroni’s post-hoc multiple comparison tests were performed. These tests were used to identify groups with statistically significant differences between them (*p* < 0.05). Similarly, interactions between breed and the SNP 2 genotype (and also between breed and the SNP 3 genotype) were verified to determine whether they could significantly differentiate the results of the analyzed dependent variables (fat content and individual milk fatty acids tested).

All analyses were performed using the Statistica v.13.1 package.

## 3. Results

### 3.1. Frequency of SNPs Genotypes of the FASN Gene

The frequency of genotype occurrence for each of the SNPs studied is presented in Table 2.

Analyzing the SNP g.16024A/G, it was noticed that the GG genotype most often appeared in RW and ZR cows (frequency: 0.85 and 0.79, respectively; *p* < 0.00001), while the A/G genotype SNP g.16024A/G was characterized by the highest frequency of occurrence in RP cows (frequency:0.29; *p* < 0.00001). However, no significant differences were found between the breeds of the cows studied in relation to the frequency of the AA genotype.

The next SNP tested was SNP g.16039T/C. It was found that in RW and ZR cows, the most common genotype was the homozygous CC variant (frequency: 0.77 and 0.67, respectively; *p* = 0.0015). In the case of RP cows, it was noted that the T/C genotype was the most common (frequency:0.29; *p* = 0.0015). However, no significant differences were found in the frequency of the TT SNP g.16039T/C genotype between the studied breeds.

Analyses of SNP g.16060A/C did not show statistically significant differences in the frequency of CC, AA, and A/C genotypes between the tested breeds of cows (*p* = 0.17).

### 3.2. Effect of FASN Gene Polymorphisms on Milk Fat Content and Fatty Acid Profile

#### 3.2.1. Effect of the SNP g.16024A/G

The post-hoc Bonferroni’s tests did not show significant differences in fat concentration between the genotypes of SNP g.16024A/G of the breeds studied (Table S1).

The significant differences (*p* < 0.05) were noted only in the content of the three UFAs tested, C16:1, C18:1n9t, and C20:1, between the genotypes of SNP g.16024A/G of the tested animals (Table S1).

**Figure 2:**
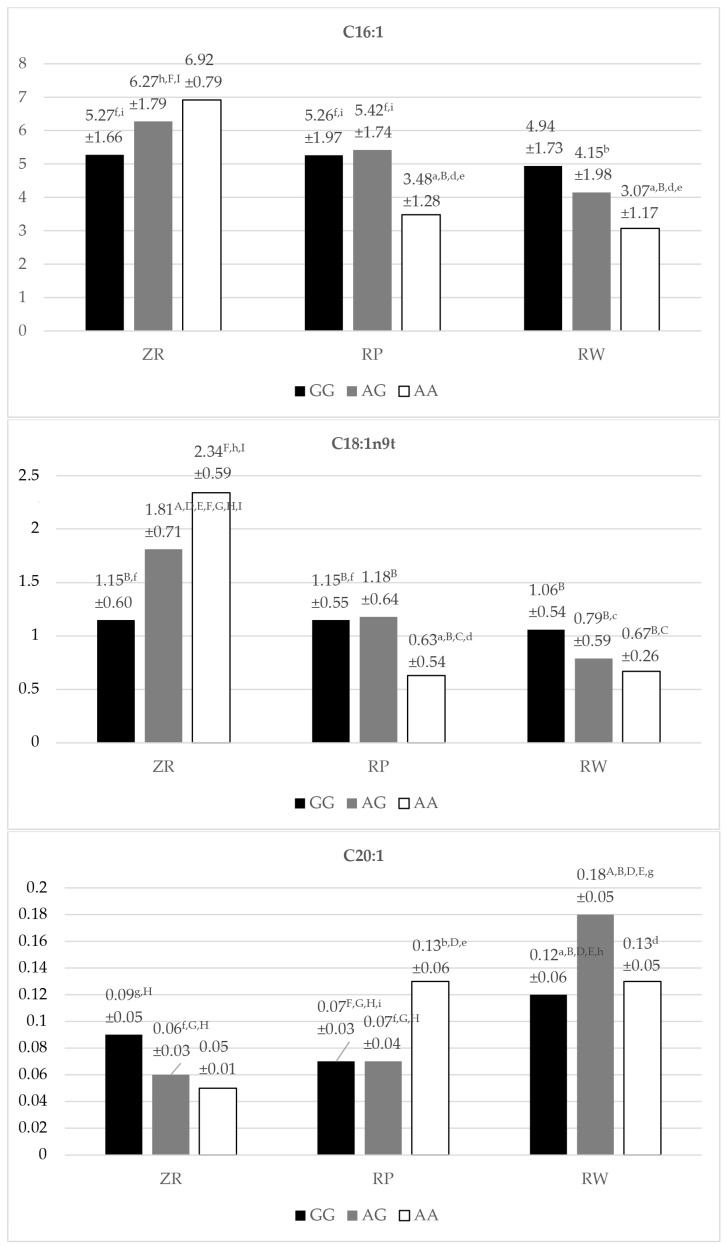
Content (mean ± SD) of C16:1, C18:1n9t, C20:1 fatty acids in the milk of Polish Red-White, Polish Red, and Polish Holstein-Friesian Red-White cows depending on the SNP g.16024A/G (SNP 1) genotypes of the FASN gene. ZR—Polish Red-White breed; RP—Polish Red breed; RW—Polish Holstein-Friesian Red-White breed; GG, AG, AA—SNP g.16024A/G genotypes; a, b, c, d, e, f, g, h, i—values differ significantly between SNP g.16024A/G (SNP 1) genotypes within fatty acid (*p* < 0.05); A, B, C, D, E, F, G, H, I—values differ highly significantly between SNP g.16024A/G (SNP 1) genotypes within fatty acid (*p* < 0.01); a, A—GG polymorphism variant of the SNP g.16024A/G for the ZR cows; b, B—AG polymorphism variant of the SNP g.16024A/G for the ZR cows; c, C—AA polymorphism variant of the SNP g.16024A/G for the ZR cows; d, D—GG polymorphism variant of the SNP g.16024A/G for the RP cows; e, E—AG polymorphism variant of the SNP g.16024A/G for the RP cows; f, F—AA polymorphism variant of the SNP g.16024A/G for the RP cows; g, G—GG polymorphism variant of the SNP g.16024A/G for the RW cows; h, H—AG polymorphism variant of the SNP g.16024A/G for the RW cows; i, I—AA polymorphism variant of the SNP g.16024A/G for the RW cows; SD—standard deviation.

The highest concentration of C16:1 acid (palmitoleic acid) was found in the milk fat of AA homozygotes of the ZR breed (6.92%) (Figure 2). Heterozygotes A/G (6.27%) of the ZR breed were characterized by significantly higher amounts of this acid in comparison with A/G heterozygotes of the RW (*p* < 0.05) breed and AA homozygotes (*p* < 0.01) of the RW and RP breeds. GG homozygotes (5.27%) of the ZR breed had significantly (*p* < 0.05) more C16:1 acid in the milk fat compared to the amount of this acid in the milk fat of AA homozygotes of the RW and RP breeds. Similarly, A/G heterozygotes (5.42%) of the RP breed had statistically significantly (*p* < 0.05) more C16:1 acid in the tested milk compared to the milk of AA homozygotes of the RW and RP breeds. Moreover, significantly (*p* < 0.05) higher amounts of C16:1 acid were found in the homozygotes of the GG SNP g.16024A/G variant of the RP breed (5.26%) than in the homozygotes of the AA variant of the mentioned SNP of the RW and RP breeds. The lowest amount of C16:1 acid was found in the milk fat (3.07%) of the homozygous AA variant of the RW cows.

Another fatty acid, the concentration of which differed statistically significantly (*p* < 0.05, *p* < 0.01) between the studied cattle breeds, was an acid from the MUFA group: C18:1n9t (elaidic acid) (Figure 2). Similarly to C16:1 acid, the highest content of C18:1n9t acid was found in the milk fat of ZR cows with the AA genotype variant (2.34%), displaying a significantly higher concentration than in the milk of AA homozygotes (*p* < 0.01) of the RW and RP breeds and in the milk of A/G heterozygotes (*p* < 0.05) of the RW breed. It turned out that the A/G genotypic variant (1.81%) of the ZR breed had a significantly (*p* < 0.01) higher amount of C:18:1n9t acid in the milk fat compared to as many as seven genotypes of SNP g.16024A/G of the FASN gene among all the cow breeds studied. It was also noted that the GG homozygotes (1.15%) of the ZR and RP breeds had a significantly (*p* < 0.05) higher amount of elaidic acid than the AA genotypic variant (0.63%) of the RP breed (the lowest concentration of this acid in milk fat among the examined cow breeds). An equally low content of this acid was recorded for the RW breed in the AA (0.67%) and A/G (0.79%) genotype variants.

In the case of C20:1acid (eicosenoic acid), the highest amount was found in A/G heterozygotes of the RW breed (0.18%) (Figure 2). Milk from cows of the A/G genotype variant had a significantly (*p* < 0.01) higher content of this acid compared to the five genotypes of the cattle breeds studied. Statistically significantly (*p* < 0.05, *p* < 0.01) higher levels of C20:1 acid were found in the milk of AA homozygous cows (0.13%) of both the RP and RW breeds in relation to some polymorphic variants of the studied conservative breeds—ZR and RP (Table 2). The lowest amount of eicosenic acid was found in the milk fat of ZR cows with the AA genotype variant (0.05%).

#### 3.2.2. Effect of the SNP g.16039T/C

Bonferroni’s post-hoc tests did not show statistically significant differences in the amount of milk fat between the genotypes of SNP g.16039T/C of the breeds studied (Table S2).

On the other hand, significant differences (*p* < 0.05) were noted in the contents of some of the studied SFAs—C10:0, C12:0, C14:0, C16:0, and C18:0—and UFAs—C18:1n9c (classified as MUFA, monounsaturated fatty acid) and C18:2n6c (classified as PUFA, polyunsaturated fatty acid)—as well as in the total number of tested SFAs and UFAs between the genotypes of the tested animals (Table S2).

**Figure 3:**
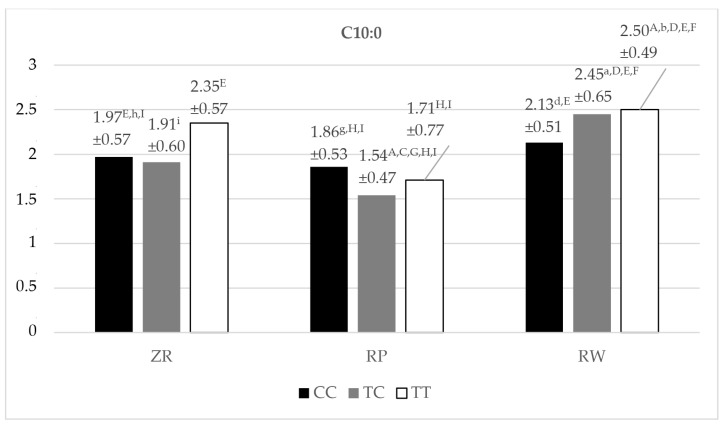

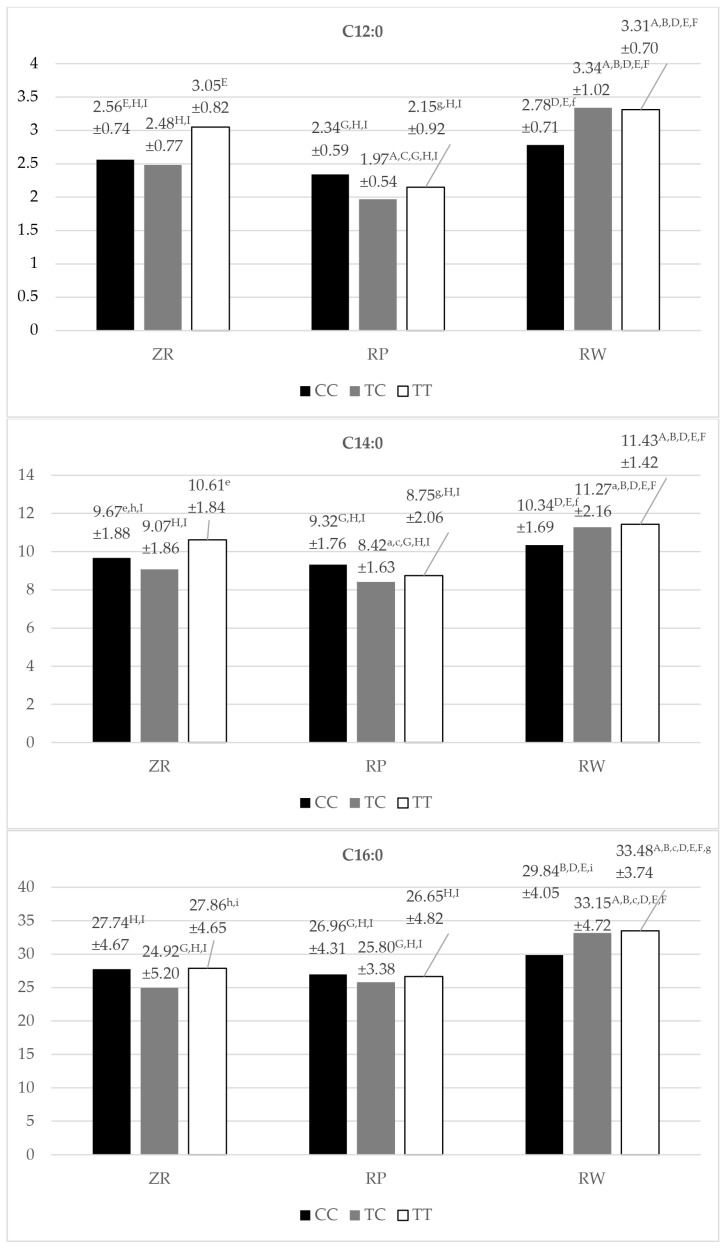

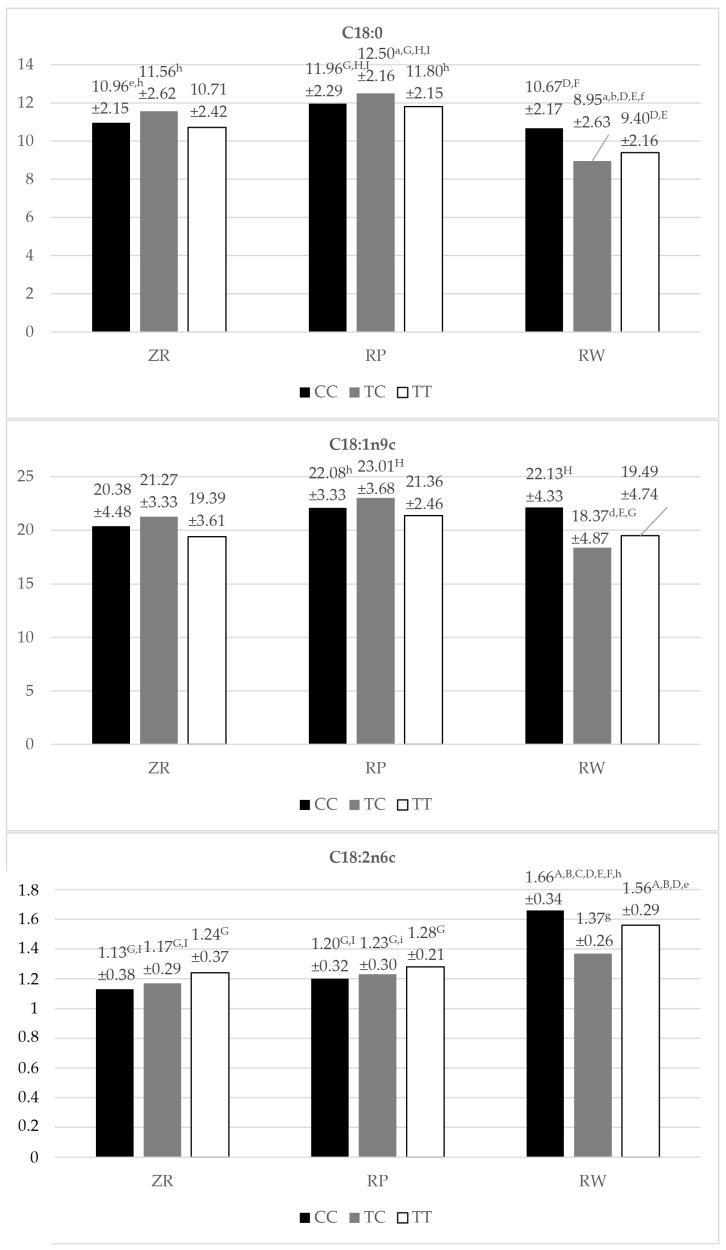

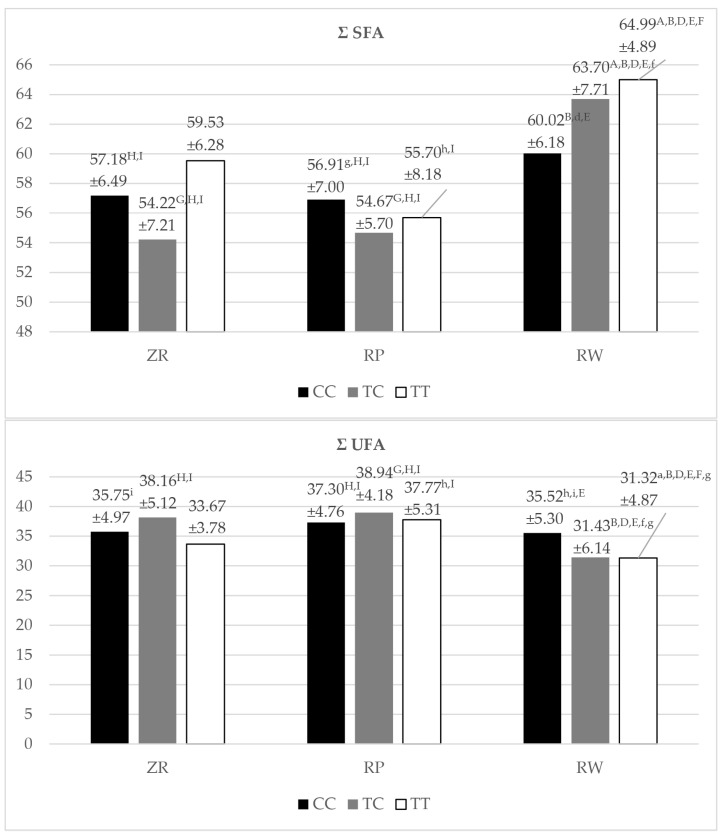
Content (mean ± SD) of C10:0, C12:0, C14:0, C16:0, C18:0, C18:1n9c, C18:2n6c fatty acids and the total content of SFA acids and UFA acids in the milk of Polish Red-White, Polish Red, and Polish Holstein-Friesian Red-White cows depending on the SNP g.16039T/C (SNP 2) genotype of the FASN gene. ZR—Polish Red-White breed; RP—Polish Red breed; RW—Polish Holstein-Friesian Red-White breed; CC, TC, TT–SNP g.16039T/C genotypes; a, b, c, d, e, f, g, h, i—values differ significantly between SNP g.16039T/C (SNP 1) genotypes within fatty acid (*p* < 0.05); A, B, C, D, E, F, G, H, I—values differ highly significantly between SNP g.16039T/C (SNP 1) genotypes within fatty acid (*p* < 0.01); a, A—CC polymorphism variant of the SNP g.16039T/C for the ZR cows; b, B—TC polymorphism variant of the SNP g.16039T/C for the ZR cows; c, C—TT polymorphism variant of the SNP g.16039T/C for the ZR cows; d, D—CC polymorphism variant of the SNP g.16039T/C for the RP cows; e, E—TC polymorphism variant of the SNP g.16039T/C for the RP cows; f, F—TT polymorphism variant of the SNP g.16039T/C for the RP cows; g, G—CC polymorphism variant of the SNP g.16039T/C for the RW cows; h, H—TC polymorphism variant of the SNP g.16039T/C for the RW cows; i, I—TT polymorphism variant of the SNP g.16039T/C for the RW cows; SD—standard deviation.

The highest amount of C10:0 acid (capric acid) was determined in milk fat for all genotypic variants of SNP g.16039T/C of the FASN gene for the RW breed: TT, 2.50%; T/C, 2.45%; and CC,2.13% (Figure 3). The TT genotype variant of the aforementioned SNP for the RW breed was statistically significantly (*p* < 0.01, *p* < 0.05) characterized by a higher content of C10:0 acid in milk fat in comparison with as many as five genotypes within the breeds ZR and RP. The heterozygousT/Cvariant of the RW breed showed significantly (*p* < 0.05, *p* < 0.01) more C10:0 acid in the milk than the content of this acid in the milk of cows for as many as four genotypes of the SNP g.16039T/C within the breeds ZR and RP. CC homozygotes of the RW breed were characterized by milkfat with a significantly higher content ofC10:0 acid compared to the CC (*p* < 0.05) and T/C (*p* < 0.01) variants of the RP breed. Statistically significant (*p* < 0.01) differences were also found in the concentration of C10:0 acid between some genotypes of the ZR and RP breeds. The lowest amounts ofC10:0 acid were found in all genotypic variants of the RP breed. The difference between the highest (2.50%) and the lowest (1.54%) concentration ofC10:0 acid in milk fat turned out to be statistically significant (*p* < 0.01).

The highest amount of C12:0 acid (lauric acid) was recorded in the milk fat of RW cows with the T/C genotype variant (3.34%). The concentration of C14:0 acid (myristic acid) was highest in TT homozygotes (11.43%) of the RW breed (Figure 3). The lowest levels of both C12:0 and C14:0 acid were found in the heterozygous T/C variant of the RP breed (1.97% and 8.42%, respectively). All these genotypes differed significantly (*p* < 0.01) regarding the content of these acids compared to as many as five other genotypes of the tested breeds (Figure 3). Statistically significant differences were found in the content of C12:0 acid between the fat of the homozygote CC (2.78%) of the RW breed and the homozygotes CC (2.34%, *p* < 0.01) and TT (2.15%, *p* < 0.05) of the RP breed. In the case of C14:0 acid, the same rule of occurrence of statistically significant (*p* < 0.01) differences in its concentration between the genotypes of the SNPg.16039T/C of the FASN gene was observed as described above for C12:0 acid. In addition, statistically significantly (*p* < 0.05, *p* < 0.01) more C14:0 acid was noted in the milk fat of the heterozygous genotype T/C of the RW breed than in the milk fat of specific polymorphic variants of the ZR and RP breeds. T/C heterozygotes of the RP breed had a significantly lower content of C14:0 acid in the fat of their milk compared to the CC and TT homozygotes of the ZR breed (*p* < 0.05) and when compared with all polymorphic variants of the RW breed (*p* < 0.01). As a result of this study, statistically significant differences in the concentration of C14:0 acid were found when comparing the milk fat of the RW cows (CC variant) with the milk fat of the RP cows (with the CC variant, *p* < 0.01, and with the TT variant, *p* < 0.05).

The highest concentration of C16:0 acid (palmitic acid) was found in the milk fat of all genotypic variants of the SNP g.16039T/Cfor RW cows. It turned out that TT homozygotes of the RW breed were characterized by a significantly (*p* < 0.05, *p* < 0.01) higher concentration of this acid compared to as many as seven other genotypes of the SNP g.16039T/C of the studied breeds (Figure 3). The T/C heterozygous genotype of RW cows differed significantly (*p* < 0.01, *p* < 0.05) in the content of C16:0 acid in their milk fat compared with as many as six genotypes of the studied animal breeds within the breeds ZR and RP. In the case of CC homozygotes in RW cows, a number of statistically significant differences (*p* < 0.01, *p* < 0.05) in the content of C16:0 acid were also noted in comparison with other genotypes within the studied cattle breeds. The lowest content of C16:0 acid was determined in the milk fat of T/C heterozygotes (24.92%) of the ZR breed. The difference between the highest and lowest amount of C16:0 acid in the fat of the tested milk was found to be highly statistically significant (*p* < 0.01).

In the case of C18:0 acid (stearic acid), its highest content was observed in all genotypic variants of the RP breed (Figure 3). The genotypic variants CC and T/C SNP g.16039T/C of the FASN gene for cattle belonging to the RP breed were characterized by statistically significant (*p* < 0.01) higher concentration of this acid compared to its amount in the milk fat of all genotypic variants in RW cows. All the genotypic variants of the RW breed had the lowest C18:0 acid content among the genotypes of the studied breeds. The T/C SNP g.16039T/C genotype of the RP cows was also significantly (*p* < 0.05) characterized by a higher amount of C18:0 acid than in the case of the milk fat of the CC homozygotes of the ZR cows. A significantly (*p* < 0.05) lower C18:0 acid level was found for CC homozygotes of the ZR breed when comparing its content in milk fat with that of T/C heterozygotes of the RP breed.

One of the two UFAs for which statistically significant differences in its amount were noted between the genotypes of the studied cattle breeds was C:18:1n9c acid (cis 9 oleic acid), classified as an MUFA (Figure 3). The highest amount of C:18:1n9c acid was found in the milk fat of the genotypic variant T/C (23.01%) of the RP breed (containing significantly (*p* < 0.01) more of this acid than the fat of T/C heterozygotes (18.37%) of the RW breed, with the lowest amount of C:18:1n9c acid among all genotypes of the studied breeds. The milk fat of RW cows with the genotype T/C showed significant differences in the concentration of cis 9 oleic acid compared with two other genotypes of the studied breeds: CC (*p* < 0.01) of the RW breed and CC (*p* < 0.05) of the RP breed.

The second UFA for which statistically significant differences in its content were found between the genotypes of the studied cattle breeds was C18:2n6c acid (linoleic acid), belonging to the group of PUFAs (Figure 3). The highest amount of this acid was determined in the milk fat of cows with all genotypic variants of the RW breed. The lowest linoleic acid content was found in the milk fat of ZR cows with the CC polymorphic variant (1.13%). It is worth noting that the homozygous variant CC (1.66%) of the RW breed, characterized by the highest content of C:18:2n6c acid in the fat of the studied milk, had statistically significantly (*p* < 0.01, *p* < 0.05) more of this acid in comparison with as many as seven genotypes of the tested breeds (a definite predominance of *p* < 0.01). The homozygous polymorphism TT SNP g.16039T/C of the RW breed (the second genotype in terms of the amount of linoleic acid) was characterized by fat with a significantly (*p* < 0.05, *p* < 0.01) higher content of linoleic acid compared to as many as four genotype variants within the studied cattle breeds (Figure 3).

The RW breed had the highest content of the total amount of all SFAs studied in all its genotypic variants (Figure 3). It is worth mentioning that the homozygous genotypes of CC of the RP breed were characterized by a statistically significantly (*p* < 0.05, *p* < 0.01) lower sum of all SFAs tested than the polymorphic variants of the aforementioned SNP of the RW cows: CC, T/C, and TT. It was found that both T/C heterozygotes and TT homozygotes for the RW breed were additionally characterized by a statistically significantly higher total amount of studiedSFAs in their milk fat than the CC homozygotes (*p* < 0.01) of the ZR breed and TT homozygotes (*p* < 0.05 in relation to the T/C homozygotes of the RW breed and *p* < 0.01 in relation to the TT homozygotes of the RW breed, respectively) of the RP breed. The lowest percentage content of all the SFAs tested was shown by the T/C genotype of the ZR breed. This content was significantly (*p* < 0.01) lower compared to the highest concentration of the total amount of studiedSFAs (all genotypic variants of the RW breed: CC, T/C and TT).

The highest content of the total amount of all studied UFAs was recorded for T/C heterozygotes of SNP g.16039T/C of the RP breed (38.94%) (Figure 3). Equally high concentrations were determined for T/C heterozygous polymorphism of the ZR breed and TT and CC homozygotes of the RP breed. It turned out that the lowest total content of tested UFAsin the studied milk was characterized by both TT homozygotes (31.32%) and T/C heterozygotes (31.43%) of SNP g.16039T/C of the RW breed. It should be noted that these genotypic variants of the RW breed showed a number of significant differences (*p* < 0.05, *p* < 0.01; predominantly *p* < 0.01) in the content of the total amount of UFAs determined in the studied milk fat between six (for the TT variant of the RW breed) and five (for the T/C variant of the RW breed) genotypes of the studied breeds. A highly significant (*p* < 0.01) difference was noted in the summed concentration of all UFAs studied in milk fat between the highest (T/C of the RP breed) and the lowest (TT and T/C of the RW breed) contents.

#### 3.2.3. Effect of the SNP g.16060A/C

Bonferroni’s post-hoc tests showed no significant differences in milk fat concentration between the genotypes of SNP g.16060A/C in cows of the breeds studied (Table S3).

The significant differences (*p* < 0.05) were noted in the content of two SFAs, C18:0 and C20:0, and one UFA (classified as an MUFA), C18:1n9t, between the genotypes of SNP g.16060A/C of the FASN gene of the tested animals (Table S3).

**Figure 4:**
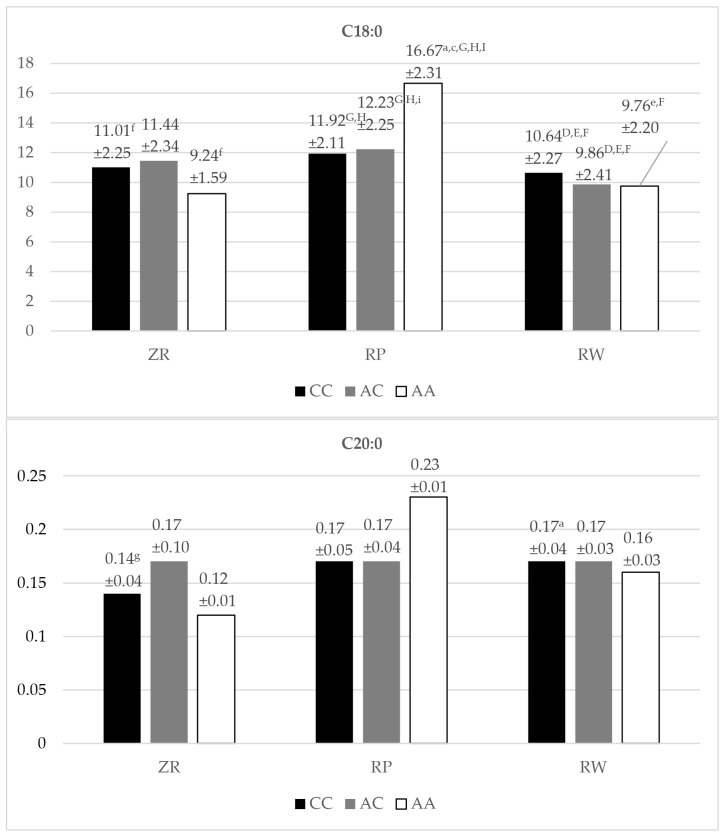

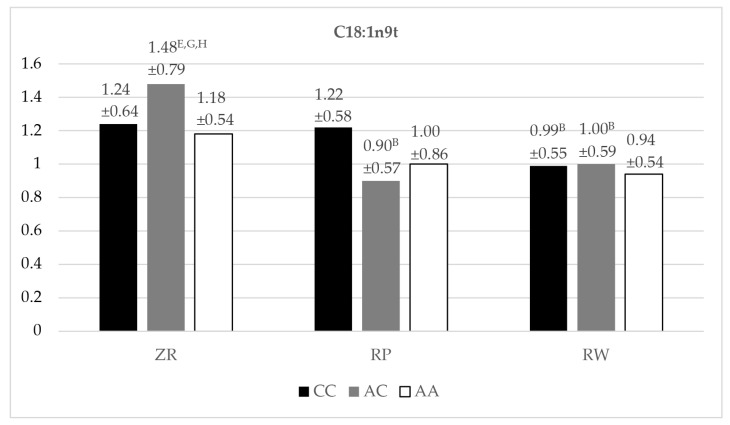
Content (mean ± SD) of C18:0, C20:0, C18:1n9t fatty acids in the milk of Polish Red-White, Polish Red, and Polish Holstein-Friesian Red-White cows depending on the SNP g.16060A/C (SNP 3) genotype of the FASN gene. ZR—Polish Red-White breed; RP—Polish Red breed; RW—Polish Holstein-Friesian Red-White breed; CC, AC, AA—SNP g.16060A/C genotypes; a, c, e, f, g, i—values differ significantly between SNP g.16060A/C (SNP 3) genotypes within fatty acid (*p* < 0.05); B, D, E, F, G, H, I—values differ highly significantly between SNP g.16060A/C (SNP 3) genotypes within fatty acid (*p* < 0.01); a—CC polymorphism variant of the SNP g.16060A/C for the ZR cows; B—AC polymorphism variant of the SNP g.16060A/C for the ZR cows; c—AA polymorphism variant of the SNP g.16060A/C for the ZR cows; D—CC polymorphism variant of the SNP g.16060A/C for the RP cows; e, E—AC polymorphism variant of the SNP g.16060A/C for the RP cows; f, F—AA polymorphism variant of the SNP g.16060A/C for the RP cows; g, G—CC polymorphism variant of the SNP g.16060A/C for the RW cows; H—AC polymorphism variant of the SNP g.16060A/C for the RW cows; i, I—AA polymorphism variant of the SNP g.16060A/C for the RW cows; SD—standard deviation.

The highest amount of C18:0 acid (stearic acid) was determined in the milk fat of the RP cows with the AA genotype variant; there was a statistically significantly (*p* < 0.05, *p* < 0.01) higher concentration of this acid in the tested milk fat compared to as many as five genotypes in the cattle breeds ZR and RW (Figure 4). The A/C heterozygous genotype of the RP breed was significantly (*p* < 0.01, *p* < 0.05) associated with a higher concentration of stearic acid in comparison with all genotypic variants of the RW breed. It was also found that the genotypic variant CC of the RP breed had a significantly (*p* < 0.01) higher content of C18:0 acid in the fat of the milk tested than the concentration of this acid in the milk fat of the CC and A/C variants of the RW breed. The lowest stearic acid was determined for AA homozygotes of the RP breed. The difference between the highest and lowest amounts of C18:0 acid was found to be statistically significant (*p* < 0.05).

The highest amount of C20:0 acid (eicosanic acid) was determined in the milk of AA homozygotes (0.23%) of the RP breed, while the lowest was found in the milk of AA homozygotes (0.12%) of the ZR breed (Figure 4). Significant differences (*p* < 0.05) were noted in the amount of this acid in the milk fat only between CC homozygotes of the ZR breed and CC homozygotes of the RW breed.

The highest concentration of C18:1n9t acid (elaidic acid) was found in A/C heterozygotes of the ZR breed (1.48%) (Figure 4). Moreover, the tendency to increase the content of C18:1n9t in milk fat was observed in the following order: the highest was observed for CC homozygotes (1.24%) of the ZR breed, the next highest was observed for CC homozygotes (1.22%) of the RP breed, finally followed by AA homozygotes (1.18%) of the ZR breed. The genotypic variant A/C for the ZR breed turned out to have a statistically (*p* < 0.01, *p* < 0.05) higher concentration of C18:1n9t acid than the three specified genotypic variants of SNP g.16060A/C within the RW and RP breeds (Figure 4). The lowest amount of elaideic acid was found in the milk fat of the RP cows (genotype: A/C)—0.90%. The difference between the highest and lowest amounts of elaidic acid was found to be highly statistically significant (*p* < 0.01).

The presented research results allowed us to conclude that the milk of cows of conservative breeds, ZR and RP, more often had a more favorable fatty acid profile than milk of cows of the RW breed directed to unilateral dairy use. It was concluded that all tested breeds (ZR, RP, and RW) were characterized by high homozygosity values (*p* < 0.01 for SNP g.16024A/G and g.16039T/C and *p* = 0.17 for SNP g.16060A/C), thus suggesting low genetic diversity within them. However, heterozygosity is an important factor for estimating the genetic variability of animals [32].

## 4. Discussion

FASN is a candidate gene influencing the fat content and specific fatty acid composition in milk and animal meat [15,19,22,33].

Otto et al. [22] and Motoyama et al. [25] define the FASN gene as one of the lipogenic genes responsible for fat accumulation and fatty acid metabolism in both the meat and adipose tissue of animals.

### 4.1. Effect of the SNP g.16024A/G

SNP g.16024A/G is a nonsynonymous mutation [34] located in the 34th exon of the FASN gene. This mutation determines the nonconservative mutation of threonine to alanine [28,35].

Schennik et al. [29] presented the relationship between SNP g.16024A/G and the concentration of C14:0 (myristic) and C18:2n6 (linoleic) acids in the milk of Dutch Holstein-Friesian cows. Theirsearch, described in this article, confirms this statement, but for the milk fat of cows of the following breeds: ZR, RP, and RW. In all breeds of the studied cattle, the amount of myristic acid was higher than the content of linoleic acid. Similarly to the cattle studied by Schennik et al. [29], AA homozygotes of RW and RP cattle were characterized by the highest amounts of C14:0 acid (10.94% and 9.69%, respectively), along with the highest content of C18:2n6 acid (1.79% and 1.30%, respectively). For the ZR breed (the second conservative breed studied), the highest concentration of these acids was observed for GG homozygotes (C14:0: 9.74% and C18:2n6: 1.19%). This variant of the SNP g.16024A/Gpolymorphism was the genotype most commonly observed for the ZR breed (0.79). The AA genotype was the least frequently appearing genotype within the RW (0.06) and RP (0.11) breeds.

Barton et al. [24] also reported the high frequency of the GG genotype variant (0.60) SNP g.16024A/G of the FASN gene in the studied population of Fleckvieh bulls (dairy and meat breed of the Simmental type). The polymorphic variant AA (0.05) was found to be the variant with the lowest frequency of occurrence. Barton et al. [24] found a significant relationship between SNP g.16024A/G of the FASN gene and the concentration of such SFAs as C14:0 and C16:0 and the MUFA C18:1n9 (cis-9) in the muscles and adipose tissue of a subcutaneous population of Fleckvieh bulls.

GG homozygotes of Chinese Holstein cows (0.76) [34] and Slovak Holstein cattle (0.68) [36] were also the most frequent genotypic variants of SNP g.16024A/G. Our own research confirms the GG polymorphism as that with the highest frequency of appearance in the following breeds: ZR (0.79), RP (0.60), and RW (0.85). The most similar frequency of occurrence of the GG genotype to Chinese Holstein cows was recorded for ZR cows, while in the case of cattle studied by Miluchova et al. [36], RP cows displayed the most similar frequency of occurrence. The results we recorded regarding the frequency of the AA homozygous genotype for the ZR, RP, and RW breeds are consistent with the observations of Miluchova et al. [36] and at the same time contradictory with the analyses of Kawaguchi et al., who observed the highest frequency of the AA genotype for Japanese Black Cattle [37]. However, this genotype variant was the least common both in the study by Miluchova et al. [36] (0.02) and in our own research (0.02 for ZR, 0.11 for RP, 0.06 for RW). It is worth noting that the frequency of AA homozygotes was identical for Slovak Holstein cattle and for the ZR breed and amounted to 0.02. An equally low frequency of appearance of AA homozygotes (0.01) was characterized by cows studied by Zhou et al. [34].

The genotypic variant AA SNP g.16024A/G was characterized by the lowest content of milk fat for all studied cattle breeds: ZR, RP, and RW. Similarly, Zhou et al. [34] found the lowest amount of fat in the milk of Chinese Holstein cows with the AA homozygous variant. In contrast, AA homozygotes of Slovak Holstein cattle [23] were associated with the highest amount of fat in their milk. An analogy to the results of the study by Miluchova et al. [23] was found for a higher fat concentration in A/G heterozygotes of the SNP g.16024A/G of the FASN gene in comparison with the amount of GG fat homozygotes in cows of the RW breed and the conservative breeds ZR and RP. Mauriæ et al. [38], in their studies involving Simmental cattle and Holstein x Simmental hybrids, noted a similar correlation, with a higher fat content for A/G heterozygotes than for AA homozygotes. An analogous regularity regarding the highest concentration of fat in heterozygous A/G cows, while at the same time with the lowest amount of fat in AA homozygote milk, was found by Zhou et al. [34] in Chinese Holstein cows. It is worth mentioning that the GG polymorphic variant of the SNP g.16024A/G of the FASN gene in the studied cows belonging to the RP breed was characterized by a very similar amount (difference of 0.01%) of milk fat compared to the polymorphic variant A/G of cows of this breed. An analogy was noted for the GG and A/G genotypic variants of SNP g.16024A/G (0.01% difference) in Chinese Holstein cows [34].

As Roy et al. [19] and Matsumoto et al. [27] pointed out, the G allele of the FASN gene causes an increase in the amount of fat in cow’s milk. The above statement is not supported by the study presented by Čitek et al. [39], according to which the A allele of the FASN gene increases the fat content in the milk of Czech Simmental and Holstein cows (including their hybrids) kept in the Czech Republic.

According to Miluchova et al. [23], SNP 16024A/G can be used as a kind of genetic marker in breeding work to improve the quality of milk (and dairy products) and its nutritional value (due to the fat content and fatty acid composition, among other factors). Further research in this area is necessary [23].

Matsuhashi et al. [35] did not record a correlation between SNP g.16024A/G and the amount of subcutaneous fat in Japanese Black cattle meat.

### 4.2. Effect of the SNP g.16039T/C and SNP g.16024A/G

SNP g.16039T/C, like SNP g.16024A/G of the FASN gene, is a nonsynonymous mutation located in the 34th exon affecting the nonconserved mutations of tryptophan (W) to arginine (R) and threonine (T) to alanine (A), respectively. Both of these mutations are located close to each other [21,24,26,27,28,35]. Due to such a close location of both polymorphisms, the symbols of the encoded amino acids TW and AR (the so-called haplotypes), which correspond to the alleles of the studied polymorphisms, are often used in the literature [26,27,28,40].

Abe et al. [28] and Walker et al. [40] recorded the highest frequency of GG genotypes (for SNP g.16024A/G) and CC (for SNP g.16039T/C) for the tested breeds. In the case of Abe et al. [28], these highest frequencies were recorded for the three breeds studied, Holstein (0.70), Angus (0.97), and Hereford (0.86), and in the case of Walker et al. [40], they were recorded for the breeds Nelore (0.97), Gir (0.92), and Pantaneira (0.88) kept in Brazil. Meanwhile, in the case of Mauric et al. [26], they were recorded for the following breeds: Simmental (0.71) and Holstein x Simmental (0.73). Our own research confirms the highest frequency of the GG genotype for SNP g.16024A/G and the highest frequency of the CC genotype for SNP g.16039T/C for the following breeds: ZR, RP, and RW. This statement is also true for SNP g.16024A/G, according to research conducted by Miluchova et al. [36]. Abe et al. [28] noted that the most common genotype for Japanese Black cattle (0.47) was the AA variant (for SNP g.16024A/G) and the TT variant (for SNP g.16039T/C). The same conclusions were drawn by Kawaguchi et al. [37] for SNP g.16024A/G, who also studied Black Japanese cattle. Walker et al. [40], in their study on cattle bred in Brazil, also noted for the Wagyu breed the significantly high frequency (*p* < 0.01) of the appearance of the genotypic variants AA and TT, respectively.

Mauric et al. [26], on the other hand, recorded a significantly low incidence of AA and TT homozygotes (Simmental cattle: 0.01; Holstein crosses: 0.00; *p* < 0.05). In the research work presented here, the lowest frequency of the genotypic variants AA SNP g.16024A/G and TT SNP g. 16039T/C for cattle of the following breeds was confirmed: ZR, RP, and RW.

Mauric et al. [26] noted a statistically significant (*p* < 0.05) relationship of the heterozygotes A/G and T/C of the studied SNPs of the FASN gene with the highest fat concentration in the milk of Holstein hybrids. The results of our own research confirm the highest fat content for heterozygous variants of SNP g.16024A/G in all tested cow breeds—ZR, RP, and RW—and for T/C heterozygotes of SNP g.16039T/C of the RW breed. The identical fat concentration (4.47%) was observed in the milk of RW cows (genotype: A/G SNP g.16024A/G) and in the milk of Holstein crosses (genotype: A/G SNP g.16024A/G) [26].

In Simmental cattle, Mauric et al. [26] found the highest fat content for GG and CC homozygotes of the SNPs g.16024A/G and g.16039T/C of the FASN gene, respectively. Similarly, Matsumoto et al. [27] pointed to higher fat concentrations for the aforementioned homozygous polymorphisms GG and CC for Holstein cattle. An analogous relationship was noted in our own studies for the CC homozygous variants of the SNP g.16039T/Cin ZR cows. Once again, similar amounts of milk fat were observed: Simmental breed—4.11% [26]; ZR breed—3.99% (own research). In the case of RP cows, the genotypic variant TT SNP g.16039T/C was associated with the highest content of milk fat.

Our own research results for the ZR breed are confirmed by the study of Abe et al. [28], including Black Japanese cattle. An correlation analogous to Abe et al. [28] can be noted regarding the AA variant of SNP g.16024A/G, correlated with a decrease in the concentration of C14:0, C14:1, and C16:0 acids and thus with an increase in the amount of C18:0 acid in the tested milk fat (in Abe [28] in the dorsal and intermuscular fat of the tested cattle). In the case of the TT SNP g.16039T/C genotype in the milk of ZR cows, a similar result to Abe et al. [28] was found only with regard to the relationship with a reduced amount of C16:1 acid (in Abe [28] in the dorsal and intermuscular fat of the examined cattle).The opposite analogy to the relationship presented in the research by Abe et al. [28] was also noticed. The TT SNP g.16039T/C variant resulted in the highest content of C14:0 and C16:0 acids and the lowest content of C18:0 and C18:1 acids. The AA SNP g.16024A/G variant for the RP breed decreased the amount of C14:1 and C16:1 (*p* < 0.05) acids and increased the amount of C14:0, C16:0, C18:0, and C18:1 acids in milk fat. TT SNP g.16039T/C homozygotes for the RP breed were correlated differently than in the research by Abe et al. [28], with lower concentrations of C18:0 and C18:1 acids and higher concentrations of C14:0, C14:1, C16:0, and C16:1 acids. AA SNP g.16024A/G homozygotes of the RW breed were characterized by a lower concentration of C14:1, C16:0, and C16:1 acids and simultaneously a higher amount of C14:0, C18:0, and C18:1 acids. TT SNP g.16039T/C homozygotes of the RW breed were characterized by a reduced content of C18:0 and C16:1 acids and a higher content of C14:0 and C16:0 acids.

Mauric et al. [26], in their studies on milk, found that the variants of the GG SNP g.16024A/G and CC SNP g.16039T/C of the FASN gene in the studied Holstein hybrids are correlated with the lowest concentration of the acids C14:0 (similarly in Abe et al. [28] for the AA and TT genotype variants, respectively) and C16:0 (*p* < 0.05) (similarly in the own research for SNP g.16024A/G for the RW breed), and at the same time with the highest contents of C14:1 (similarly in our own research for SNP g.16024A/G for ZR breed) and C18:1n9 (*p* < 0.05) (similarly in Abe et al. [28] for C18:1 acid for the genotypes AA and TT and similarly in our own research for C18:1n9c acid for SNP g.16024A/G for the ZR breed and C18:1n9t acid for SNP g.16024A/G for the RW breed, *p* < 0.01) compared to all genotypes of the abovementioned SNPs within both Holstein hybrids as well as Simmental cows.

It is worth mentioning that the heterozygotes for both of the aforementioned SNPs of the FASN gene in Holstein hybrids were associated with the lowest content of C16:1 acid [26].

When looking at the GG and CC polymorphisms of the SNPs in the Holstein hybrids studied by Mauric et al. [26], it should be emphasized that they correlated significantly (*p* < 0.05) with the highest content of an acid important for human health, C18:3n3, and with the lowest concentration of the total amount of SFAs tested (unfavorable in the human diet) and with the highest content of the health-promoting MUFAs studied. A similarity to the above associations was noted in the author’s own studies (but only for the SNP g.16024A/G). In the case of the aforementioned SNP of the FASN gene, the GG genotype variant of the RW breed was characterized by the highest concentration of C18:3n3 acid and the highest amount of such MUFAs as C16:1 and C18:1n9t (*p* < 0.01) and the sum of other tested trans acids from the C18:1 group. The GG SNP g.16024A/G homozygotes of the RW breed were characterized by the lowest concentration of all SFAs analyzed.

The literature and our own studies show that SNP g.16039T/C and SNP g.16024A/G of the FASN gene significantly define, above all, the high content of C18:1 acid both in meat (Japanese Black cattle [28]) and in milk (ZR, RP and RW breeds—own research, *p* < 0.05, *p* < 0.01; Holstein crossbreeds [26], *p* < 0.05). This is a feature desired by consumers due to the more nutritious nature of the products consumed, namely meat and milk.

Based on the discussed associations of the SNPs g.16024A/G and g.16039T/C of the FASN gene with the content of specific fatty acids, it is concluded (similar to Matsumoto [27]) that the mentioned SNPs (and their specific genotypic variants) may be a source of information about estimating the composition of fatty acids in milk and its products, and thus, they can be treated as genetic markers used for improving milk quality [27].

### 4.3. Effect of the SNP g.16060A/C

In the case of the SNP g.16060A/C of the FASN gene, no studies on this polymorphism in cattle have been found in the literature. Therefore, the associations of this hitherto unknown SNP (along with its genotypic variants) with the milk fat metabolism of the studied cattle breeds ZR, RP and RW are described in the Results (3.1, 3.2, 3.2.3) and Conclusions sections, providing innovative information and potentially encouraging other researchers to further analyze this polymorphism of the FASN gene in order to, among other things, estimate its potential and usefulness in the genetic improvement of dairy cattle.

The obtained research results should become a basis for their further development, including LD (linkage disequilibrium) analysis, not only for the alleles of SNP g.16024A/G and SNP g.16039T/C, but also for determining the degree of correlation of alleles of the previously unknown SNP g.16060A/C with alleles of the SNPs mentioned above.

## 5. Conclusions

As a result of the conducted research, it was found that the milk of ZR cows with genotypes AA and G/A SNP g.16024A/G of the FASN gene was characterized by a more favorable fatty acid profile (the highest concentration of fatty acids important for human health: C18:1n9t and C16:1) among the other examined cow breeds. Milk from these cows also had the lower fat content expected by consumers. In the case of the SNP g.16039C/T, milk from heterozygous C/T cows of the RP breed turned out to be the most beneficial for human health (significant association with the highest content of health-promoting fatty acids: C18:1n9c and with the highest amount of all total studied UFA acids). Moreover, the C/T polymorphism for the RP breed significantly determined the lowest or one of the lower levels of many SFAs tested. In the case of the SNP g.16060C/A, it was found that the milk with higher nutritional values was obtained from AA homozygotes (associated with the lowest content of some SFA acids: C18:0 and C20:0) and C/A heterozygotes (associated with the highest concentration of the health-promoting acid: C18:1n9t) in ZR cows. However, from a dietary point of view, the milk of these cows of the C/A variant was more attractive due to the lower fat content among all the analyzed genotype variants. Due to the fact that the tested SNPs g.16024A/G, g.16039T/C, and g.16060C/A are located close to each other, it should be borne in mind that it may turn out that only the linkage of a specific set of alleles of the tested SNPs can determine a significant effect for a specific feature, which requires further research and analysis. The described research can be treated as a preliminary study aimed at developing research on the degree of correlation of alleles of the studied SNPs and their impact on the quality of cows’ milk.

## Figures and Tables

**Table 1:** Information about the location, amino acid change, primer sequences, and annealing temperature for SNP g.16024A/G (SNP 1), SNP g.16039C/T (SNP 2), and SNP g.16060A/C (SNP 3) of the FASN gene.

SNP	Loci	Location AA Change	Primer Sequence (5′-3′)	Annealing Temperature (°C)
SNP 1	rs208645216 g.16024A/G	Exon 34 Thr/Ala	F: GAGACGCCAGGGTGTGC R: GTTGAAGATGCCTCCCACG	65 °C
SNP 2	rs209734560 g.16039C/T	Exon 34 Trp/Arg	F: AGGCAGGTCCACGAGTG R: TCTAAAGCCGTCCTCAC	65 °C
SNP 3	rs211379310 g.16060A/C	Exon 34	F: GCAGGTCCTGGTGTCCA R: TGGAGGGCTTCTTAGG	65 °C

AA Change—amino acid change.

**Table 2:** Frequencies of the SNP g.16024A/G (SNP 1), SNP g.16039T/C (SNP 2), and SNP g.16060A/C genotypes for the Polish Red-White breed, Polish Red breed, and Polish Holstein-Friesian Red-White breed in the FASN gene.

Breed	*n*	SNP 1 GENOTYPES			*n*	SNP 2 GENOTYPES			*n*	SNP 3 GENOTYPES		
ZR	94	GG	AG	AA	93	CC	TC	TT	94	CC	AC	AA
		79% *	19%	2%		67% *	22%	12%		68%	29%	3%
RP	146	GG	AG	AA	147	CC	TC	TT	147	CC	AC	AA
		60%	29% *	11%		57%	29% *	14%		66%	32%	2%
RW	226	GG	AG	AA	240	CC	TC	TT	241	CC	AC	AA
		85% *	8%	6%		77% *	13%	10%		70%	24%	6%
		χ^2^ = 36.25 df = 4 *p* < 0.00001 V_C_ = 0.20				χ^2^ = 17.59 df = 4 *p* = 0.0015 V_C_ = 0.14				χ^2^ = 6.37 df = 4 *p* = 0.17		

ZR—Polish Red-White breed; RP—Polish Red breed; RW—Polish Holstein-Friesian Red-White breed; n—number of animals; SNP 1—single nucleotide polymorphism g.16024A/G; SNP 2—single nucleotide polymorphism g.16039T/C; SNP 3—single nucleotide polymorphism g.16060A/C; *—values differ highly significantly between the SNP g.16024A/G (SNP 1), SNP g.16039T/C (SNP 2), and SNP g.16060A/C genotypes within rows (*p* < 0.01) (frequencies of genotypes considered for each SNP separately); χ^2^—Pearson’s χ^2^ test; df—number of degrees of freedom; Vc—Cramér’s V coefficient.

## Data Availability

The database relating to the presented study and confirming the presented research results is not publicly available for data privacy reasons. A request to share data regarding the described study can be sent to paulinaprzybylska2018@gmail.com.

## Supplementary Materials

The following supporting information can be downloaded at: https://www.mdpi.com/article/10.3390/ani14131934/s1, Table S1: Content (mean ± SD) of fat and fatty acids in the milk of Polish Red-White, Polish Red and Polish Holstein-Friesian Red-White cows depending on the SNP g.16024A/G (SNP 1) genotypes of the FASN gene; Table S2: Content (mean ± SD) of fat and fatty acids in the milk of Polish Red-White, Polish Red, and Polish Holstein-Friesian Red-Whitecows depending on the SNP g.16039T/C (SNP 2) genotype of the FASN gene; Table S3: Content (mean ± SD) of fat and fatty acids in the milk of Polish Red-White, Polish Red and Polish Holstein-Friesian Red-White cows depending on the SNP g.16060A/C (SNP 3) genotype of the FASN gene.
