# Supplementary material for: Relationship between Selected SNPs (g.16024A/G, g.16039T/C and g.16060A/C) of the FASN Gene and the Fat Content and Fatty Acid Profile in the Milk of Three Breeds of Cows
Source: Animals (Basel). 2024 Jun 29;14(13):1934. doi: 10.3390/ani14131934 (PMC11240365; doi:10.3390/ani14131934)
Supplement: Supplementary file 1 [file animals-14-01934-s001.zip › Table_S1.pdf]

Table S1. Content (mean ± SD) of fat and fatty acids in the milk of Polish Red-White, Polish Red and Polish Holstein-Friesian Red-White cows depending on the SNP g.16024A/G (SNP 1) genotypes of the FASN gene.

Trait (%)	ZR Genotype			RP Genotype			RW Genotype		
	**GG** (*n*=74)	**AG** (*n*=18)	**AA** (*n*=2)	**GG** (*n*=88)	**AG** (*n*=42)	**AA** (*n*=16)	**GG** (*n*=193)	**AG** (*n*=19)	**AA** (*n*=14)
Fat	3.88 ±0.85	4.05 ±0.67	3.08 ±1.13	4.45 ±0.93	4.58 ±0.94	4.44 ±0.81	4.20 ±0.83	4.47 ±0.86	4.01 ±0.94
C4:0	0.73 ±0.40	0.60 ±0.37	0.67 ±0.28	0.69 ±0.38	0.74 ±0.41	1.01 ±0.41	0.69 ±0.30	0.85 ±0.33	0.98 ±0.26
C6:0	0.91 ±0.32	0.82 ±0.29	0.79 ±0.16	0.83 ±0.31	0.86 ±0.30	1.10 ±0.29	0.90 ±0.24	0.99 ±0.21	1.08 ±0.20
C8:0	0.76 ±0.21	0.70 ±0.21	0.63 ±0.09	0.67 ±0.21	0.66 ±0.20	0.81 ±0.20	0.79 ±0.17	0.79 ±0.15	0.85 ±0.16
C10:0	2.03 ±0.58	1.94 ±0.63	1.50 ±0.18	1.74 ±0.57	1.66 ±0.53	2.01 ±0.54	2.19 ±0.55	2.11 ±0.51	2.32 ±0.57
C11:0	0.16 ±0.06	0.18 ±0.06	0.12 ±0.01	0.05 ±0.02	nm nm	0.05 ±0.02	0.30 ±0.78	0.08 ±0.08	nm nm
C12:0	2.62 ±0.76	2.56 ±0.83	1.96 ±0.10	2.22 ±0.67	2.09 ±0.60	2.41 ±0.58	2.90 ±0.81	2.75 ±0.75	3.00 ±0.81
C13:0	0.07 ±0.03	0.08 ±0.06	nm nm	0.06 ±0.03	0.06 ±0.01	0.07 ±0.03	0.11 ±0.06	0.12 ±0.07	0.10 ±0.04
C14:0	9.74 ±1.93	9.44 ±1.80	8.08 ±0.11	8.92 ±1.80	8.82 ±1.89	9.69 ±1.49	10.54 ±1.78	10.10 ±2.06	10.94 ±1.87
C15:0	1.10 ±0.23	1.22 ±0.26	0.95 ±0.20	1.20 ±0.24	1.25 ±0.27	1.18 ±0.15	1.21 ±0.32	1.37 ±0.43	1.16 ±0.17
C16:0	27.60 ±5.05	25.54 ±3.90	24.77 ±1.02	26.61 ±4.33	26.10 ±3.67	27.86 ±4.06	30.38 ±4.40	31.27 ±5.20	30.50 ±3.62
C17:0	0.67 ±0.18	0.66 ±0.13	0.73 ±0.01	0.77 ±0.15	0.79 ±0.14	0.73 ±0.12	0.67 ±0.15	0.71 ±0.10	0.60 ±0.08
C18:0	11.00 ±2.31	11.27 ±2.33	13.05 ±0.63	11.83 ±2.32	12.16 ±2.02	13.21 ±2.11	10.14 ±2.28	10.81 ±2.78	10.67 ±1.47
C20:0	0.15 ±0.05	0.17 ±0.09	nm nm	0.16 ±0.05	0.17 ±0.04	0.19 ±0.04	0.17 ±0.04	0.18 ±0.05	0.16 ±0.04
Σ SFA	57.38 ±6.93	55.10 ±5.83	53.23 ±2.40	55.67 ±6.70	55.32 ±6.64	60.22 ±6.96	60.37 ±6.47	61.94 ±7.56	62.32 ±5.97
C14:1	1.42 ±0.95	1.36 ±0.28	0.82 ±0.05	1.24 ±0.24	1.33 ±0.28	1.27 ±0.17	1.44 ±0.31	1.45 ±0.38	1.43 ±0.28
C16:1	5.27[f,i] ±1.66	6.27[h,F,I] ±1.79	6.92 ±0.79	5.26[f,i] ±1.97	5.42[f,i] ±1.74	3.48[a,B,d,e] ±1.28	4.94 ±1.73	4.15[b] ±1.98	3.07[a,B,d,e] ±1.17
C17:1	0.37 ±0.12	0.37 ±0.09	0.30 ±0.11	0.46 ±0.09	0.47 ±0.12	0.47 ±0.11	0.33 ±0.13	0.39 ±0.12	0.29 ±0.16
C18:1n9c	20.84 ±4.40	19.11 ±2.84	20.27 ±2.40	22.24 ±3.63	22.02 ±2.98	23.04 ±3.12	21.44 ±4.73	21.49 ±5.01	23.45 ±4.42
C18:1n8c (11c)	0.78 ±0.31	0.70 ±0.14	0.67 ±0.12	0.83 ±0.26	0.80 ±0.23	0.81 ±0.23	0.96 ±0.32	0.93 ±0.46	1.05 ±0.25
C18:1n9t	1.15[B,f] ±0.60	1.81[A,D,E,F,G,H,I] ±0.71	2.34[F,h,I] ±0.59	1.15[B,f] ±0.55	1.18[B] ±0.64	0.63[a,B,C,d] ±0.54	1.06[B] ±0.54	0.79[B,c] ±0.59	0.67[B,C] ±0.26
C18:1n7t	2.86 ±1.37	3.10 ±1.00	2.89 ±0.52	3.12 ±1.52	3.35 ±1.52	2.85 ±1.04	1.76 ±0.98	1.94 ±1.16	1.57 ±0.42
other *trans* 18:1	0.38 ±0.31	0.27 ±0.08	0.28 ±0.01	0.26 ±0.11	0.23 ±0.06	0.27 ±0.03	0.43 ±0.20	0.42 ±0.09	0.59 ±0.44

C18:2n6c	1.19 ±0.35	1.02 ±0.34	1.13 ±0.01	1.23 ±0.30	1.17 ±0.30	1.30 ±0.27	1.64 ±0.33	1.37 ±0.33	1.79 ±0.26
CLA	1.15 ±0.60	1.25 ±0.54	0.83 ±0.11	1.23 ±0.68	1.30 ±0.60	1.01 ±0.52	0.61 ±0.30	0.58 ±0.23	0.59 ±0.18
C18:3n3	0.76 ±0.26	0.86 ±0.30	0.98 ±0.09	0.90 ±0.28	0.90 ±0.21	0.85 ±0.21	0.49 ±0.22	0.48 ±0.16	0.38 ±0.10
C20:1	$0.09^{g,H}$ ±0.05	$0.06^{f,G,H}$ ±0.03	0.05 ±0.01	$0.07^{F,G,H,i}$ ±0.03	$0.07^{f,G,H}$ ±0.04	$0.13^{b,D,e}$ ±0.06	$0.12^{a,B,D,E,h}$ ±0.06	$0.18^{A,B,D,E,g}$ ±0.05	0.13^{d} ±0.05
C20:4n6	0.09 ±0.05	0.09 ±0.06	nm nm	0.08 ±0.03	0.08 ±0.03	0.10 ±0.03	0.12 ±0.04	0.13 ±0.05	0.15 ±0.04
C20:5n(*cis*-5,8,11,14,17)	0.07 ±0.03	0.07 ±0.01	nm nm	0.07 ±0.02	0.08 ±0.03	0.11 ±0.05	0.06 ±0.02	0.10 ±0.02	nm nm
Σ UFA	36.06 ±5.34	35.86 ±3.68	36.72 ±0.52	37.98 ±4.84	38.25 ±4.35	36.13 ±4.79	34.93 ±5.67	33.96 ±6.28	34.99 ±5.50
SFA+UFA	93.44 ±2.81	90.96 ±2.70	89.95 ±1.88	93.65 ±2.76	93.56 ±3.04	96.36 ±2.79	94.50 ±7.94	95.90 ±2.08	97.31 ±1.33

SFAs—saturated fatty acids; UFAs—unsaturated fatty acids; ZR—Polish Red-White breed; RP—Polish Red breed; RW—Polish Holstein-Friesian Red-White breed; n—number of animals; a, b, c, d, e, f, g, h, i—values differ significantly between SNP g.16024A/G (SNP 1) genotypes within rows ($p<0.05$); A, B, C, D, E, F, G, H, I—values differ highly significantly between SNP g.16024A/G (SNP 1) genotypes within rows ($p<0.01$); p—probability; a, A—GG polymorphism variant of the SNP g.16024A/G for the ZR cows; b, B—AGpolymorphism variant of the SNP g.16024A/G for the ZR cows; c, C—AA polymorphism variant of the SNP g.16024A/G for the ZR cows; d, D—GG polymorphism variant of the SNP g.16024A/G for the RP cows; e, E—AGpolymorphism variant of the SNP g.16024A/G for the RP cows; f, F—AA polymorphism variant of the SNP g.16024A/G for the RP cows; g, G—GG polymorphism variant of the SNP g.16024A/G for the RW cows; h, H—AG polymorphism variant of the SNP g.16024A/G for the RW cows; i, I—AA polymorphism variant of the SNP g.16024A/G for the RW cows; nm—not marked; SD—standard deviation.
