# Supplementary material for: Relationship between Selected SNPs (g.16024A/G, g.16039T/C and g.16060A/C) of the FASN Gene and the Fat Content and Fatty Acid Profile in the Milk of Three Breeds of Cows
Source: Animals (Basel). 2024 Jun 29;14(13):1934. doi: 10.3390/ani14131934 (PMC11240365; doi:10.3390/ani14131934)
Supplement: Supplementary file 1 [file animals-14-01934-s001.zip › Table_S2.pdf]

Table S2. Content (mean ± SD) of fat and fatty acids in the milk of Polish Red-White, Polish Red, and Polish Holstein-Friesian Red-Whitecows depending on the SNP g.16039T/C (SNP 2) genotype of the FASN gene.

Trait (%)	ZR Genotype			RP Genotype			RW Genotype		
	**CC** (*n*=62)	**TC** (*n*=20)	**TT** (*n*=11)	**CC** (*n*=84)	**TC** (*n*=42)	**TT** (*n*=21)	**CC** (*n*=184)	**TC** (*n*=32)	**TT** (*n*=24)
Fat	3.99 ±0.82	3.48 ±0.86	4.04 ±0.52	4.55 ±0.92	4.27 ±0.87	4.65 ±0.92	4.23 ±0.82	4.31 ±0.91	4.12 ±0.72
C4:0	0.71 ±0.41	0.61 ±0.31	0.79 ±0.39	0.76 ±0.42	0.74 ±0.40	0.65 ±0.28	0.75 ±0.33	0.60 ±0.24	0.82 ±0.33
C6:0	0.88 ±0.33	0.84 ±0.29	1.00 ±0.28	0.90 ±0.33	0.82 ±0.28	0.83 ±0.30	0.93 ±0.25	0.89 ±0.21	1.02 ±0.26
C8:0	0.74 ±0.22	0.72 ±0.21	0.85 ±0.18	0.72 ±0.21	0.62 ±0.18	0.67 ±0.26	0.79 ±0.16	0.83 ±0.16	0.88 ±0.15
C10:0	$1.97^{E,h,I}$ ±0.57	1.91^{i} ±0.60	2.35^{E} ±0.57	$1.86^{g,H,I}$ ±0.53	$1.54^{A,C,G,H,I}$ ±0.47	$1.71^{H,I}$ ±0.77	$2.13^{d,E}$ ±0.51	$2.45^{a,D,E,F}$ ±0.65	$2.50^{A,b,D,E,F}$ ±0.49
C11:0	0.17 ±0.06	0.14 ±0.05	0.20 ±0.07	0.04 ±0.02	0.03 ±0.01	0.08 ±0.02	0.29 ±0.75	0.03 ±0.01	0.09 ±0.09
C12:0	$2.56^{E,H,I}$ ±0.74	$2.48^{H,I}$ ±0.77	3.05^{E} ±0.82	$2.34^{G,H,I}$ ±0.59	$1.97^{A,C,G,H,I}$ ±0.54	$2.15^{g,H,I}$ ±0.92	$2.78^{D,E,f}$ ±0.71	$3.34^{A,B,D,E,F}$ ±1.02	$3.31^{A,B,D,E,F}$ ±0.70
C13:0	0.07 ±0.03	0.07 ±0.02	0.09 ±0.07	0.06 ±0.02	0.06 ±0.02	0.06 ±0.03	0.10 ±0.06	0.06 ±0.01	0.12 ±0.06
C14:0	$9.67^{e,h,I}$ ±1.88	$9.07^{H,I}$ ±1.86	10.61^{e} ±1.84	$9.32^{G,H,I}$ ±1.76	$8.42^{a,c,G,H,I}$ ±1.63	$8.75^{g,H,I}$ ±2.06	$10.34^{D,E,f}$ ±1.69	$11.27^{a,B,D,E,F}$ ±2.16	$11.43^{A,B,D,E,F}$ ±1.42
C15:0	1.07 ±0.23	1.16 ±0.15	1.28 ±0.31	1.16 ±0.22	1.24 ±0.25	1.41 ±0.20	1.16 ±0.31	1.42 ±0.35	1.35 ±0.28
C16:0	$27.74^{H,I}$ ±4.67	$24.92^{G,H,I}$ ±5.20	$27.86^{h,i}$ ±4.65	$26.96^{G,H,I}$ ±4.31	$25.80^{G,H,I}$ ±3.38	$26.65^{H,I}$ ±4.82	$29.84^{B,D,E,i}$ ±4.05	$33.15^{A,B,c,D,E,F}$ ±4.72	$33.48^{A,B,c,D,E,F,g}$ ±3.74
C17:0	0.64 ±0.15	0.70 ±0.17	0.73 ±0.23	0.73 ±0.13	0.81 ±0.11	0.84 ±0.20	0.66 ±0.15	0.72 ±0.14	0.68 ±0.14
C18:0	$10.96^{e,h}$ ±2.15	11.56^{h} ±2.62	10.71 ±2.42	$11.96^{G,H,I}$ ±2.29	$12.50^{a,G,H,I}$ ±2.16	11.80^{h} ±2.15	$10.67^{D,F}$ ±2.17	$8.95^{a,b,D,E,f}$ ±2.63	$9.40^{D,E}$ ±2.16
C20:0	0.15 ±0.07	0.15 ±0.05	0.14 ±0.03	0.16 ±0.05	0.18 ±0.04	0.19 ±0.04	0.17 ±0.04	0.18 ±0.05	0.17 ±0.04
Σ SFA	$57.18^{H,I}$ ±6.49	$54.22^{G,H,I}$ ±7.21	59.53 ±6.28	$56.91^{g,H,I}$ ±7.00	$54.67^{G,H,I}$ ±5.70	$55.70^{h,I}$ ±8.18	$60.02^{B,d,E}$ ±6.18	$63.70^{A,B,D,E,f}$ ±7.71	$64.99^{A,B,D,E,F}$ ±4.89
C14:1	1.27 ±0.34	1.71 ±1.71	1.50 ±0.33	1.23 ±0.23	1.29 ±0.25	1.40 ±0.28	1.42 ±0.30	1.56 ±0.38	1.49 ±0.27
C16:1	5.62 ±1.70	5.60 ±1.76	4.93 ±1.73	4.98 ±2.05	5.24 ±1.69	5.22 ±1.85	4.58 ±1.74	5.34 ±2.16	4.42 ±1.89
C17:1	0.37 ±0.12	0.39 ±0.10	0.31 ±0.07	0.46 ±0.10	0.48 ±0.10	0.45 ±0.10	0.34 ±0.12	0.38 ±0.11	0.26 ±0.12
C18:1n9c	20.38 ±4.48	21.27 ±3.33	19.39 ±3.61	22.08^{h} ±3.33	23.01^{H} ±3.68	21.36 ±2.46	22.13^{H} ±4.33	$18.37^{d,E,G}$ ±4.87	19.49 ±4.74
C18:1n8c (11c)	0.79 ±0.33	0.73 ±0.14	0.69 ±0.16	0.82 ±0.27	0.82 ±0.22	0.79 ±0.15	0.98 ±0.31	0.77 ±0.35	0.85 ±0.31
C18:1n9t	1.36 ±0.68	1.33 ±0.72	1.06 ±0.65	1.03 ±0.58	1.18 ±0.60	1.24 ±0.66	1.03 ±0.56	0.81 ±0.53	0.78 ±0.45
C18:1n7t	2.76 ±1.28	3.37 ±1.43	2.79 ±1.00	3.11 ±1.65	3.20 ±1.34	3.34 ±0.65	1.91 ±0.99	1.57 ±1.02	1.32 ±0.53
other *trans*18:1	0.36 ±0.34	0.36 ±0,14	0.27 ±0,07	0.25 ±0,07	0.27 ±0,13	0.25 ±0,05	0.44 ±0,20	0.27 ±0,10	0.48 ±0.38

C18:2n6c	1.13^{G,I} ±0.38	1.17^{G,I} ±0.29	1.24^{G} ±0.37	1.20^{G,I} ±0.32	1.23^{G,i} ±0.30	1.28^{G} ±0.21	1.66^{A,B,C,D,E,F,h} ±0.34	1.37^{g} ±0.26	1.56^{A,B,D,e} ±0.29
CLA	1.08 ±0.56	1.39 ±0.60	1.18 ±0.62	1.20 ±0.71	1.23 ±0.56	1.36 ±0.44	0.64 ±0.29	0.62 ±0.39	0.52 ±0.18
C18:3n3	0.75 ±0.26	0.86 ±0.26	0.78 ±0.31	0.86 ±0.25	0.94 ±0.23	0.99 ±0.28	0.50 ±0.23	0.57 ±0.22	0.45 ±0.15
C20:1	0.08 ±0.05	0.07 ±0.03	0.08 ±0.04	0.08 ±0.05	0.08 ±0.05	0.06 ±0.02	0.13 ±0.06	0.13 ±0.05	0.15 ±0.04
C20:4n6	0.09 ±0.04	0.08 ±0.06	0.11 ±0.07	0.08 ±0.04	0.08 ±0.03	0.09 ±0.03	0.12 ±0.04	0.12 ±0.05	0.16 ±0.04
C20:5n (*cis*-5,8,11,14,17)	0.06 ±0.02	0.08 ±0.03	0.07 ±0.01	0.07 ±0.04	0.08 ±0.02	0.07 ±0.02	0.06 ±0.02	0.08 ±0.04	0.06 ±0.03
Σ UFA	35.75^{i} ±4.97	38.16^{H,I} ±5.12	33.67 ±3.78	37.30^{H,I} ±4.76	38.94^{G,H,I} ±4.18	37.77^{h,I} ±5.31	35.52^{h,i,E} ±5.30	31.43^{B,D,E,f,g} ±6.14	31.32^{a,B,D,E,F,g} ±4.87
SFA+UFA	92.93 ±2.93	92.38 ±2.97	93.20 ±3.21	94.20 ±3.09	93.61 ±2.60	93.47 ±3.19	94.71 ±8.09	95.12 ±2.46	96.32 ±1.79

SFAs—saturated fatty acids; UFAs—unsaturated fatty acids; ZR—Polish Red-White breed; RP—Polish Red breed; RW—Polish Holstein-Friesian Red-White breed; n—number of animals; a, b, c, d, e, f, g, h, i—values differ significantly between SNP g.16039T/C (SNP 1) genotypes within rows ($p<0.05$); A, B, C, D, E, F, G, H, I—values differ highly significantly between SNP g.16039T/C (SNP 1) genotypes within rows ($p<0.01$); p—probability; a, A—CC polymorphism variant of the SNP g.16039T/Cfor the ZR cows; b, B—TC polymorphism variant of the SNP g.16039T/Cfor the ZR cows; c, C—TT polymorphism variant of the SNP g.16039T/C for the ZR cows; d, D—CC polymorphism variant of the SNP g.16039T/C for the RP cows; e, E—TC polymorphism variant of the SNP g.16039T/C for the RP cows; f, F—TT polymorphism variant of the SNP g.16039T/C for the RP cows; g, G—CC polymorphism variant of the SNP g.16039T/C for the RW cows; h, H—TC polymorphism variant of the SNP g.16039T/C for the RW cows; i, I—TT polymorphism variant of the SNP g.16039T/C for the RW cows; SD—standard deviation
