# Supplementary material for: Relationship between Selected SNPs (g.16024A/G, g.16039T/C and g.16060A/C) of the FASN Gene and the Fat Content and Fatty Acid Profile in the Milk of Three Breeds of Cows
Source: Animals (Basel). 2024 Jun 29;14(13):1934. doi: 10.3390/ani14131934 (PMC11240365; doi:10.3390/ani14131934)
Supplement: Supplementary file 1 [file animals-14-01934-s001.zip › Table_S3.pdf]

Table S3. Content (mean ± SD) of fat and fatty acids in the milk of Polish Red-White, Polish Red and Polish Holstein-Friesian Red-White cows depending on the SNP g.16060A/C (SNP 3) genotype of the FASN gene.

Trait (%)	ZR Genotype			RP Genotype			RW Genotype		
	CC (*n*=64)	AC (*n*=27)	AA (*n*=3)	CC (*n*=97)	AC (*n*=47)	AA (*n*=3)	CC (*n*=168)	AC (*n*=58)	AA (*n*=15)
Fat	3.77	4.09	4.74	4.43	4.63	4.01	4.19	4.30	4.29
	±0.83	±0.75	±0.55	±0.94	±0.86	±0.50	±0.78	±1.31	±0.80
C4:0	0.73	0.67	0.47	0.70	0.81	0.68	0.75	0.84	0.67
	±0.40	±0.40	±0.12	±0.38	±0.43	±0.49	±0.32	±0.34	±0.30
C6:0	0.91	0.86	0.77	0.83	0.95	0.92	0.94	0.93	0.91
	±0.32	±0.32	±0.12	±0.29	±0.33	±0.46	±0.25	±0.22	±0.24
C8:0	0.76	0.73	0.73	0.65	0.75	0.72	0.80	0.78	0.82
	±0.21	±0.24	±0.14	±0.20	±0.22	±0.26	±0.17	±0.16	±0.16
C10:0	2.02	1.95	2.12	1.65	1.92	1.80	2.16	2.19	2.32
	±0.54	±0.69	±0.38	±0.54	±0.57	±0.58	±0.51	±0.70	±0.55
C11:0	0.18	0.12	nm	0.05	0.05	nm	0.27	0.05	0.12
	±0.06	±0.05	nm	±0.02	±0.02	nm	±0.72	±0.01	±0.01
C12:0	2.61	2.54	2.82	2.12	2.38	2.10	2.81	2.96	3.09
	±0.70	±0.93	±0.69	±0.63	±0.63	±0.46	±0.72	±1.15	±0.78
C13:0	0.07	0.08	0.08	0.06	0.06	0.04	0.11	0.11	0.08
	±0.03	±0.06	±0.01	±0.02	±0.02	±0.01	±0.06	±0.03	±0.03
C14:0	9.67	9.42	10.80	8.73	9.41	9.24	10.44	10.36	10.93
	±1.81	±2.08	±2.21	±1.68	±1.92	±2.35	±1.56	±2.52	±1.96
C15:0	1.10	1.13	1.28	1.25	1.15	1.24	1.20	1.26	1.22
	±0.21	±0.29	±0.07	±0.26	±0.18	±0.26	±0.30	±0.37	±0.37
C16:0	26.61	27.85	31.24	26.09	27.41	27.15	30.27	30.67	31.32
	±4.59	±5.43	±2.41	±4.14	±3.98	±6.20	±4.01	±4.43	±5.01
C17:0	0.65	0.69	0.72	0.79	0.73	0.91	0.66	0.67	0.69
	±0.16	±0.18	±0.16	±0.16	±0.10	±0.12	±0.14	±0.11	±0.15
C18:0	11.01^{f}	11.44	9.24^{f}	$11.92^{G,H}$	$12.23^{G,H,i}$	$16.67^{a,c,G,H,I}$	$10.64^{D,E,F}$	$9.86^{D,E,F}$	$9.76^{e,F}$
	±2.25	±2.34	±1.59	±2.11	±2.25	±2.31	±2.27	±2.41	±2.20
C20:0	0.14^{g}	0.17	0.12	0.17	0.17	0.23	0.17^{a}	0.17	0.16
	±0.04	±0.10	±0.01	±0.05	±0.04	±0.01	±0.04	±0.03	±0.03
Σ SFA	56.34	57.47	60.31	54.92	57.92	61.64	60.65	60.59	61.79
	±6.49	±7.35	±5.49	±6.49	±6.81	±12.71	±5.95	±7.21	±7.50
C14:1	1.46	1.22	1.34	1.29	1.24	1.03	1.43	1.45	1.48
	±1.02	±0.30	±0.36	±0.26	±0.23	±0.23	±0.30	±0.33	±0.33
C16:1	5.39	5.64	6.53	5.35	4.67	3.87	4.51	4.92	4.89
	±1.59	±1.91	±2.89	±1.87	±1.94	±1.51	±1.84	±2.03	±1.66
C17:1	0.37	0.37	0.42	0.46	0.47	0.48	0.33	0.34	0.35
	±0.10	±0.14	±0.16	±0.09	±0.12	±0.01	±0.13	±0.15	±0.11
C18:1n9c	20.73	20.42	18.85	22.28	22.34	20.64	21.58	21.88	21.06
	±4.34	±4.45	±0.37	±3.42	±3.32	±3.84	±4.05	±6.06	±5.53
C18:1n8c(11c)	0.78	0.75	0.69	0.80	0.85	0.76	0.96	0.94	0.89
	±0.31	±0.23	±0.11	±0.23	±0.28	±0.18	±0.29	±0.35	±0.38
C18:1n9t	1.24	$1.48^{E,G,H}$	1.18	1.22	0.90^{B}	1.00	0.99^{B}	1.00^{B}	0.94
	±0.64	±0.79	±0.54	±0.58	±0.57	±0.86	±0.55	±0.59	±0.54
C18:1n7t	3.11	2.54	1.54	3.26	3.00	3.15	1.97	1.60	1.44
	±1.27	±1.24	±1.02	±1.44	±1.53	±1.14	±1.03	±1.00	±0.63
other *trans*18:1	0.38	0.30	0.23	0.25	0.26	0.25	0.43	0.37	0.45
	±0.33	±0.12	±0.13	±0.07	±0.12	±0.01	±0.19	±0.14	±0.31

C18:2n6c	1.16 ±0.35	1.19 ±0.39	0.96 ±0.31	1.24 ±0.29	1.19 ±0.31	1.15 ±0.04	1.64 ±0.35	1.58 ±0.28	1.58 ±0.31
CLA	1.26 ±0.60	0.96 ±0.49	0.64 ±0.35	1.32 ±0.66	1.08 ±0.57	0.88 ±0.79	0.65 ±0.31	0.55 ±0.18	0.58 ±0.26
C18:3n3	0.78 ±0.25	0.79 ±0.31	0.68 ±0.21	0.93 ±0.28	0.84 ±0.19	0.98 ±0.18	0.51 ±0.25	0.44 ±0.10	0.52 ±0.18
C20:1	0.08 ±0.05	0.08 ±0.03	0.06 ±0.02	0.07 ±0.04	0.10 ±0.05	0.06 ±0.01	0.12 ±0.05	0.19 ±0.06	0.14 ±0.05
C20:4n6	0.09 ±0.05	0.09 ±0.04	0.07 ±0.01	0.08 ±0.03	0.09 ±0.03	0.08 ±0.01	0.12 ±0.04	0.13 ±0.04	0.11 ±0.05
C20:5n (*cis*-5,8,11,14,17)	0.07 ±0.03	0.07 ±0.02	0.06 ±0.01	0.07 ±0.03	0.08 ±0.03	0.07 ±0.01	0.07 ±0.02	nm nm	0.06 ±0.02
Σ UFA	36.51 ±4.98	35.47 ±5.28	33.09 ±2.43	38.46 ±4.55	36.91 ±4.64	34.14 ±8.74	34.90 ±5.09	35.03 ±6.83	33.87 ±6.43
SFA+UFA	92.86 ±2.90	92.94 ±3.27	93.39 ±3.07	93.38 ±2.79	94.83 ±3.02	95.78 ±3.97	94.58 ±8.70	95.62 ±2.18	95.66 ±2.10

SFAs—saturated fatty acids; UFAs—unsaturated fatty acids; ZR—Polish Red-White breed; RP—Polish Red breed; RW—Polish Holstein-Friesian Red-White breed; n—number of animals; a, c, e, f, g, i—values differ significantly between SNP g.16060A/C (SNP 3) genotypes within rows ($p<0.05$); B, D, E, F, G, H, I—values differ highly significantly between SNP g.16060A/C (SNP 3) genotypes within rows ($p<0.01$); p—probability; a—CC polymorphism variant of the SNP g.16060A/Cfor the ZR cows; B—ACpolymorphism variant of the SNP g.16060A/Cfor the ZR cows; c—AA polymorphism variant of the SNP g.16060A/Cfor the ZR cows; D—CC polymorphism variant of the SNP g.16060A/C for the RP cows; e, E—ACpolymorphism variant of the SNP g.16060A/C for the RP cows; f, F—AA polymorphism variant of the SNP g.16060A/C for the RP cows; g, G—CC polymorphism variant of the SNP g.16060A/C for the RW cows; H—AC polymorphism variant of the SNP g.16060A/C for the RW cows; i, I—AA polymorphism variant of the SNP g.16060A/C for the RW cows; nm—not marked; SD—standard deviation.
